# Missed Until Found: Diagnosis and Hysteroscopic Management of a Uterine Septum in a Woman With Primary Infertility

**DOI:** 10.7759/cureus.116093

**Published:** 2026-09-11

**Authors:** Divya Ravikumar, Shohib Shajahan, Haneen Haneefa, Malavika Pramod

**Affiliations:** 1 Department of Obstetrics and Gynaecology, Azerbaijan Medical University, Baku, AZE

**Keywords:** congenital müllerian anomaly, hysteroscopic septum resection, primary infertility, septate uterus, three-dimensional transvaginal ultrasonography

## Abstract

Congenital uterine anomalies are an important yet frequently overlooked cause of female infertility, with a septate uterus representing the most common developmental anomaly of the uterine cavity. Although some affected women remain asymptomatic, others present with infertility, recurrent pregnancy loss, or adverse obstetric outcomes, making early recognition essential for appropriate management.

We report the case of a 26-year-old nulligravid woman from Azerbaijan who presented with primary infertility of three years' duration despite regular menstrual cycles and an otherwise unremarkable infertility evaluation. Transvaginal ultrasonography followed by confirmatory imaging demonstrated a uterine septum as the underlying anatomical abnormality. After comprehensive counseling regarding available treatment options, the patient underwent successful hysteroscopic septum resection. The procedure was completed without intraoperative or postoperative complications, and follow-up evaluation confirmed restoration of a normal uterine cavity. The patient recovered well and was advised regarding future conception planning with continued reproductive follow-up.

This case highlights the importance of incorporating detailed uterine cavity assessment into the routine evaluation of women with unexplained primary infertility. It also demonstrates the effectiveness of hysteroscopic septum resection as a safe, minimally invasive procedure for restoring normal uterine anatomy. Increased clinical awareness, timely diagnosis, and access to advanced imaging modalities are essential to facilitate early intervention and optimize reproductive outcomes, particularly in resource-variable healthcare settings where congenital uterine anomalies may remain underdiagnosed.

## Introduction

Congenital uterine anomalies (CUAs) are structural abnormalities of the female reproductive tract resulting from abnormal development, fusion, or resorption of the Müllerian ducts during embryogenesis [1]. They are estimated to affect approximately 4%-7% of women in the general population, with substantially higher prevalence among women presenting with infertility, recurrent pregnancy loss, and adverse obstetric outcomes [2]. Among these anomalies, a septate uterus is the most common congenital uterine malformation and is characterized by incomplete resorption of the medial septum following normal fusion of the Müllerian ducts, resulting in a fibrous or fibromuscular partition that partially or completely divides the endometrial cavity [3].

Although many women with a septate uterus remain asymptomatic, the anomaly has been associated with infertility, recurrent miscarriage, preterm birth, fetal malpresentation, and other adverse reproductive outcomes [4,5]. The underlying mechanisms are not completely understood but are thought to involve impaired vascularization of the septal tissue, altered endometrial receptivity, and abnormal implantation, all of which may compromise embryo implantation and fetal development [5]. Given these potential reproductive consequences, accurate diagnosis and appropriate management are essential, particularly in women with otherwise unexplained infertility.

Advances in diagnostic imaging have substantially improved the evaluation of CUAs [6]. Three-dimensional transvaginal ultrasonography has emerged as the preferred first-line imaging modality because of its high diagnostic accuracy, non-invasive nature, and ability to simultaneously assess both the endometrial cavity and the external uterine contour [6,7]. Magnetic resonance imaging serves as a valuable complementary modality in complex cases or when ultrasonographic findings are inconclusive. Accurate differentiation between a septate uterus and other Müllerian anomalies, particularly a bicornuate uterus, is crucial because treatment strategies and reproductive prognoses differ considerably [8,9].

Current guidelines from the American Society for Reproductive Medicine (ASRM) and the European Society of Human Reproduction and Embryology (ESHRE) emphasize standardized classification systems and individualized management of uterine anomalies [4]. Hysteroscopic septum resection is currently considered the treatment of choice for appropriately selected patients because it is minimally invasive, associated with low complication rates, and effectively restores normal uterine cavity anatomy. While growing evidence suggests that hysteroscopic septoplasty may improve reproductive outcomes, particularly by reducing miscarriage rates, its effect on live birth rates and fertility in all women with septate uterus remains an area of ongoing investigation. Consequently, careful patient selection and individualized clinical decision-making continue to play a central role in management [8-10].

Published literature describing septate uterus from the South Caucasus region remains limited, and reports highlighting the diagnostic evaluation and minimally invasive management of this condition are scarce. This case describes a young woman from Azerbaijan who presented with primary infertility secondary to a uterine septum and was successfully managed with hysteroscopic septum resection. By integrating current evidence with regional clinical experience, this report emphasizes the importance of comprehensive anatomical assessment during infertility evaluation, timely diagnosis using contemporary imaging techniques, and appropriate surgical management to optimize future reproductive outcomes.

## Case presentation

A 26-year-old nulligravid woman from Azerbaijan presented to the Department of Obstetrics and Gynecology of Azerbaijan Medical University with a three-year history of primary infertility. She had been married for three years and had been attempting spontaneous conception throughout this period without success. Her menstrual cycles were regular, occurring every 28 days with a duration of five days and normal menstrual flow. She denied dysmenorrhea, dyspareunia, abnormal uterine bleeding, intermenstrual bleeding, postcoital bleeding, or chronic pelvic pain.

Her obstetric history was unremarkable (gravida 0, para 0), with no previous pregnancies, miscarriages, or ectopic pregnancies. She had no history of pelvic inflammatory disease, sexually transmitted infections, endometriosis, uterine surgery, or previous gynecological procedures. She had not previously undergone assisted reproductive techniques or fertility treatment. There was no history of hormonal contraceptive use immediately preceding infertility evaluation.

Her medical history was otherwise unremarkable, with no known chronic illnesses including diabetes mellitus, hypertension, thyroid disease, autoimmune disorders, or thromboembolic disease. She had no previous surgical history and was not taking any regular medications. She reported no known drug allergies. She was a non-smoker and denied alcohol consumption and recreational drug use, and her family history was negative for CUAs, infertility, recurrent pregnancy loss, or hereditary genetic disorders.

On physical examination, the patient appeared healthy and was in no acute distress. Her vital signs were stable, with a blood pressure of 125/78 mmHg, pulse rate of 73 beats/min, respiratory rate of 17 breaths/min, and body mass index of 27 kg/m². Abdominal examination was unremarkable, with no palpable masses or tenderness. Pelvic examination demonstrated normal external genitalia, a healthy cervix, and a normally sized anteverted uterus without cervical motion tenderness or adnexal masses. Bimanual examination revealed no pelvic tenderness or evidence of pelvic pathology.

As part of the infertility evaluation, routine laboratory investigations, including complete blood count, renal and liver function tests, fasting blood glucose, and serum electrolytes, were within normal limits. Hormonal assessment demonstrated normal ovarian reserve and endocrine function, including follicle-stimulating hormone (FSH), luteinizing hormone (LH), estradiol, progesterone, prolactin, thyroid-stimulating hormone (TSH), and anti-Müllerian hormone (AMH). Ovulatory function was considered normal based on hormonal evaluation and menstrual history. Semen analysis of the patient's partner demonstrated normal sperm concentration, morphology, and motility according to the World Health Organization laboratory manual for the examination and processing of human semen. Additional infertility investigations, including tubal patency assessment via hysterosalpingography (HSG), were unremarkable.

Transvaginal ultrasonography demonstrated an abnormal uterine cavity, raising suspicion for a congenital Müllerian anomaly. Subsequent three-dimensional transvaginal ultrasonography confirmed the presence of a uterine septum extending into the endometrial cavity while maintaining a normal external uterine fundal contour, consistent with a septate uterus. Both ovaries appeared morphologically normal, with preserved ovarian reserve and no evidence of endometrioma or adnexal pathology. No associated renal anomalies were identified. Based on imaging findings, the anomaly was classified according to the ASRM Müllerian Anomalies Classification (2021).

Following multidisciplinary discussion and detailed counseling regarding the diagnosis, available treatment options, expected reproductive outcomes, potential complications, and alternative management strategies, the patient elected to undergo hysteroscopic septum resection. The initial hysteroscopic findings and commencement of the operative procedure are demonstrated in Video 1.

**Video 1:** Initial hysteroscopic evaluation of the uterine cavity before septum resection. The video shows a hysteroscopic procedure to remove a uterine septum. A camera, known as a hysteroscope, is placed inside the uterus, revealing the pink lining of the uterine cavity. A thick, whitish band of tissue-the septum-is visible in the center, dividing the cavity. A surgical instrument with a yellowish loop electrode is used to carefully cut away this septal tissue. As the loop slices through the septum, you can see bubbles and a smoke-like effect, which is caused by the electrical current used to cut the tissue. The goal of this surgery is to remove the division and create a single, unified uterine cavity.

The procedure was performed under general anesthesia using a 5 mm hysteroscope. Uterine cavity distension was achieved using normal saline, and hysteroscopic evaluation confirmed the presence of a complete uterine septum extending from the uterine fundus into the endometrial cavity. Both tubal ostia were identified following complete septal resection. The septum was resected using cold scissors under direct hysteroscopic visualization until restoration of a single triangular uterine cavity was achieved. The operative time was approximately 40 minutes, with minimal estimated blood loss. The final hysteroscopic appearance following complete septum resection is shown in Video 2. No intraoperative complications, including uterine perforation, excessive bleeding, fluid overload, or cervical trauma, were encountered. Histopathological examination was not performed because the resected septal tissue was minimal and the diagnosis had already been established based on characteristic imaging and intraoperative hysteroscopic findings.

**Video 2:** Final hysteroscopic appearance following complete septum resection. This video shows the final stages and conclusion of the hysteroscopic uterine septum resection. A unified cavity: The most notable change from the beginning of the surgery is that the thick, dividing wall of tissue (the septum) has been completely cut away. The left and right sides of the uterus are now joined into a single, wide, and open cavity. Final adjustments: The surgical loop instrument is seen making final movements to smooth out the edges where the septum was attached to the uterine wall, ensuring no residual tissue blocks the cavity. Surgical debris and bubbles: The fluid inside the uterus appears somewhat cloudy with floating tissue fragments, small air bubbles, and minor bleeding. This is completely normal at the end of the procedure due to the electrosurgical cutting. The video concludes with a clear view of the restored, single uterine chamber, signaling a successful operation.

The postoperative course was uneventful. The patient remained hemodynamically stable and was discharged on postoperative day 2 with routine postoperative instructions. She received analgesics (ibuprofen) and antibiotics and was advised to avoid sexual intercourse for two weeks to allow adequate endometrial healing. Follow-up transvaginal ultrasonography performed one month after surgery demonstrated restoration of a normal uterine cavity without evidence of residual septum or intrauterine adhesions (IUAs). The patient remained asymptomatic and was counseled regarding the optimal timing of future conception, including the option of spontaneous conception and assisted reproductive techniques if clinically indicated. At the most recent follow-up, she continued routine reproductive follow-up with plans for spontaneous conception.

## Discussion

A septate uterus is the most common congenital Müllerian anomaly and results from incomplete resorption of the medial septum following normal fusion of the paired Müllerian ducts during embryogenesis [1]. Depending on the extent of resorption failure, the septum may be partial or complete, dividing the endometrial cavity while preserving a normal external uterine contour. Although many women remain asymptomatic, a septate uterus has been consistently associated with infertility, recurrent pregnancy loss, preterm birth, malpresentation, and other adverse obstetric outcomes [8]. The precise mechanism underlying impaired reproductive performance remains incompletely understood but is believed to involve poor vascularization of the septal tissue, altered endometrial receptivity, abnormal implantation, and impaired placentation, all of which may negatively influence embryo implantation and pregnancy maintenance [3,5,11]. Figure 1 schematically illustrates the normal embryological development of the Müllerian ducts and the failure of septal resorption that results in the formation of a septate uterus.

**Figure 1:**
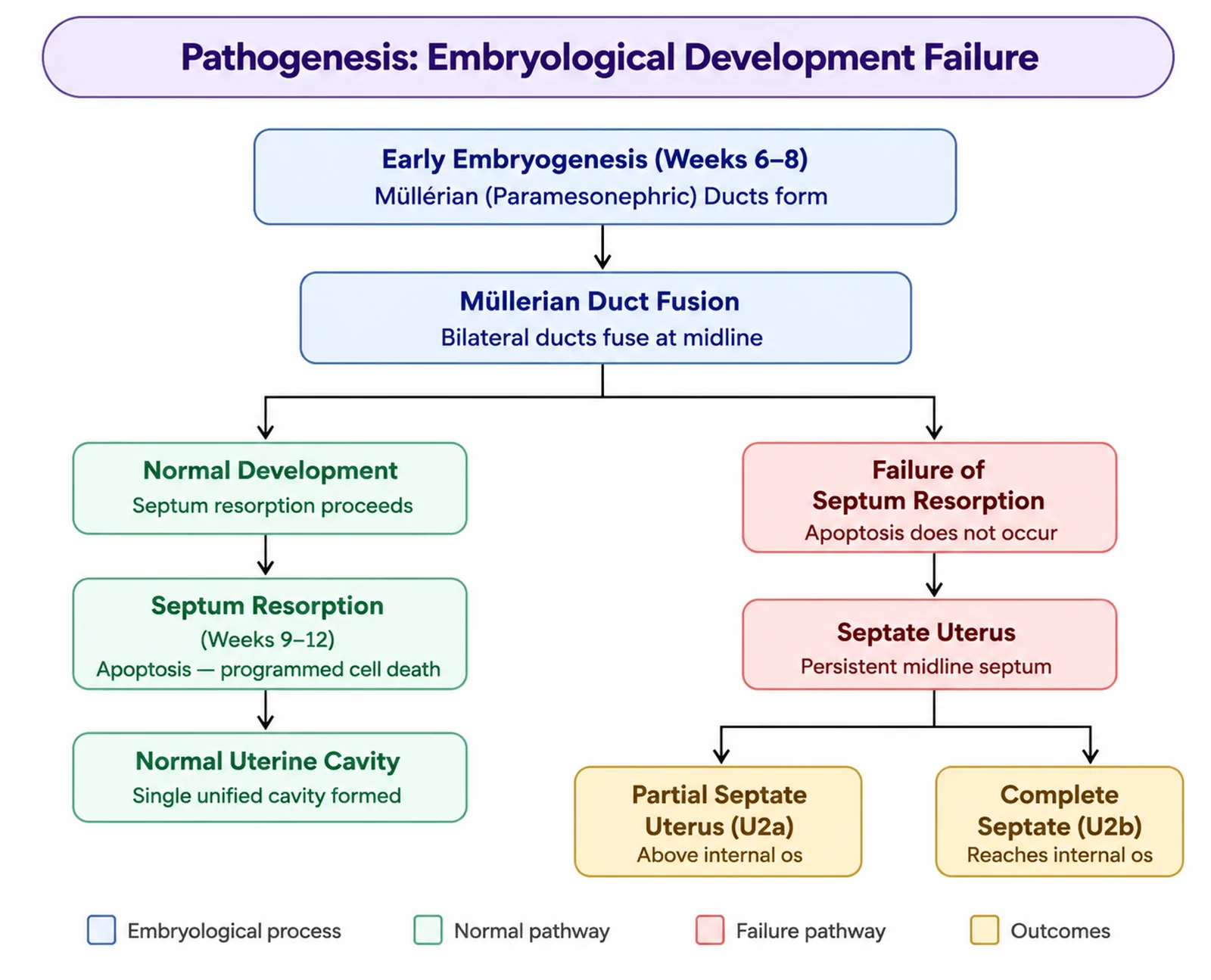
Embryological development and pathogenesis of a septate uterus. Schematic illustration showing normal fusion of the paired Müllerian ducts followed by complete resorption of the intervening septum to form a single uterine cavity. Failure of septal resorption after normal duct fusion results in the formation of a partial or complete septate uterus. The authors used Claude (Anthropic, San Francisco, CA, USA) to assist in the creation of this figure.

Accurate classification of CUAs is essential because management and reproductive prognosis vary among different malformations. The ASRM Müllerian Anomalies Classification (2021) and the ESHRE/European Society for Gynaecological Endoscopy (ESGE) classification remain the most widely accepted systems for categorizing uterine anomalies [1,2,4,10]. These standardized classifications facilitate appropriate diagnosis, improve communication among clinicians, and guide individualized management. In the present case, imaging findings were consistent with a septate uterus according to contemporary classification criteria, supporting hysteroscopic septum resection as the preferred treatment strategy [2-4]. The classification and diagnostic pathway for septate uterus, including the recommended sequence of imaging modalities and differentiation from other Müllerian anomalies, is illustrated in Figure 2.

**Figure 2:**
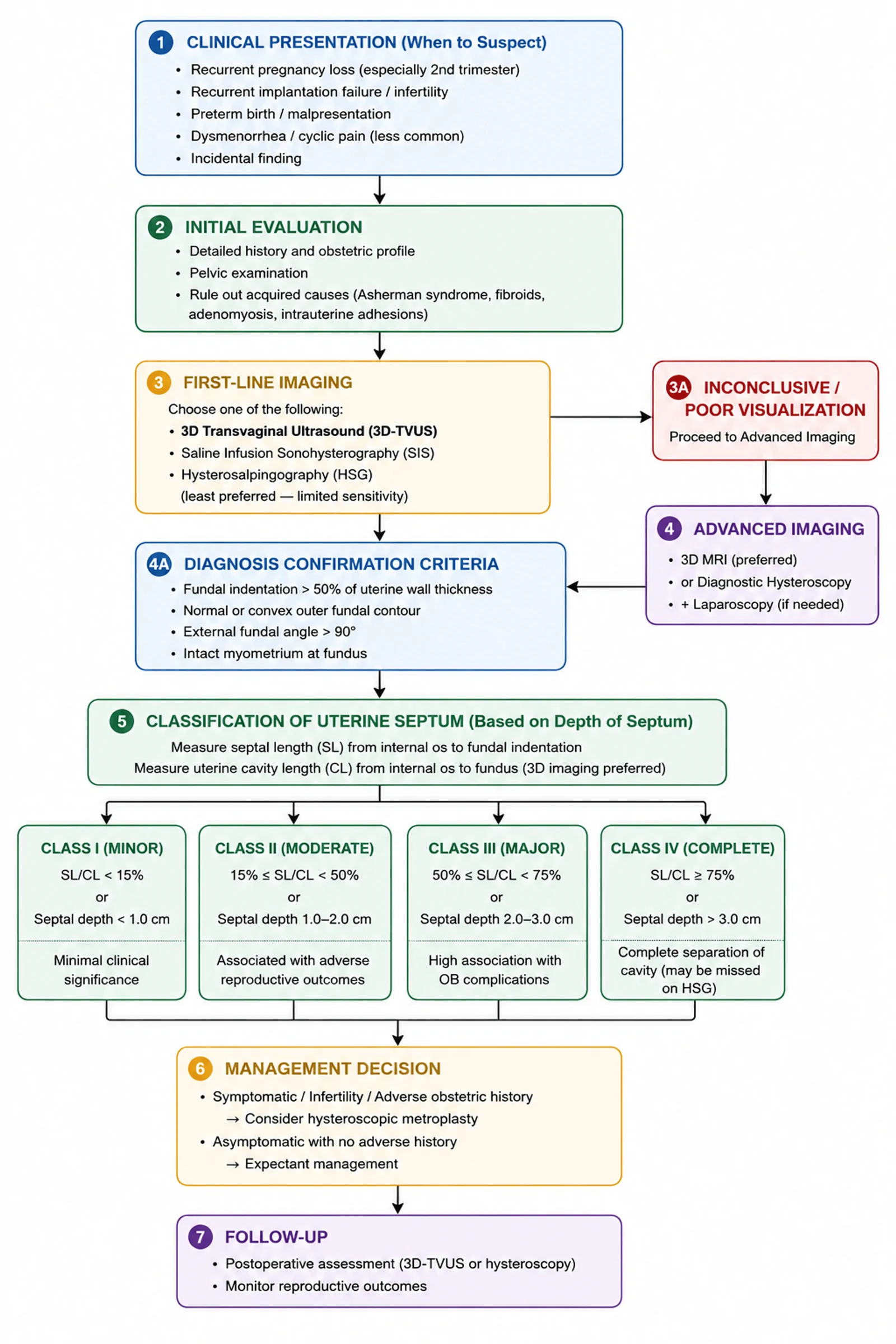
Classification and diagnostic algorithm for septate uterus. Schematic illustration summarizing the contemporary classification systems (ASRM and ESHRE/ESGE) and the recommended diagnostic approach for septate uterus, including initial clinical evaluation, 3D transvaginal ultrasonography as the preferred first-line imaging modality, complementary use of MRI in selected cases, differentiation from other Müllerian anomalies, and confirmation of the diagnosis to guide appropriate management. 1. 3D-TVUS is the preferred first-line modality. 2. Differentiate septum from bicornuate uterus: septum → normal/convex fundal contour; bicornuate → external fundal indentation. Accurate classification predicts reproductive risk and guides management. The authors used Claude (Anthropic, San Francisco, CA, USA) to assist in the creation of this figure. MRI: magnetic resonance imaging; 3D: three-dimensional; ASRM: American Society for Reproductive Medicine; ESHRE/ESGE: European Society of Human Reproduction and Embryology/European Society for Gynaecological Endoscopy

The diagnosis of a septate uterus has evolved considerably with advances in imaging technology. Three-dimensional transvaginal ultrasonography is currently regarded as the preferred first-line imaging modality because it provides excellent visualization of both the endometrial cavity and the external uterine contour while remaining non-invasive and widely accessible [12]. Figure 3 provides a schematic overview of the clinical presentation of septate uterus, highlighting its variable manifestations and associated reproductive complications.

**Figure 3:**
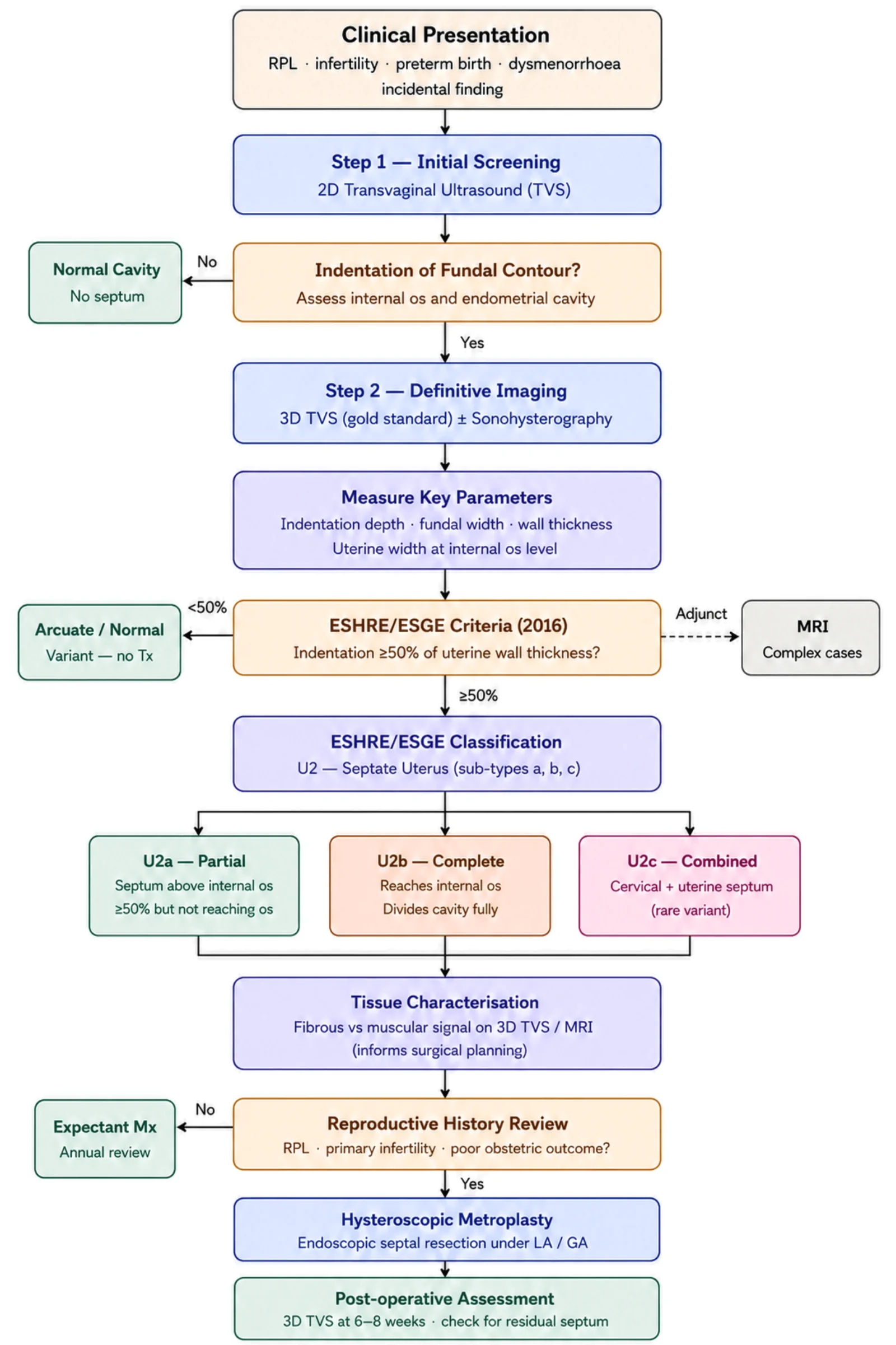
General clinical presentation of septate uterus. Schematic illustration summarizing the spectrum of clinical manifestations associated with a septate uterus. While some women remain asymptomatic, others may present with primary infertility, recurrent pregnancy loss, recurrent implantation failure, preterm birth, fetal malpresentation, abnormal uterine bleeding, dysmenorrhea, pelvic pain, or adverse obstetric outcomes. The severity and presentation vary depending on the extent of the septum and individual patient characteristics. The authors used Claude (Anthropic, San Francisco, CA, USA) to assist in the creation of this figure. RPL: recurrent pregnancy loss; Tx: treatment; ESHRE/ESGE: European Society of Human Reproduction and Embryology/European Society for Gynaecological Endoscopy; MRI: magnetic resonance imaging; Mx: medical management; LA/GA: local anesthesia/general anesthesia

Magnetic resonance imaging serves as an important complementary investigation when sonographic findings are inconclusive or complex Müllerian anomalies are suspected. Accurate differentiation between a septate uterus and other CUAs, particularly a bicornuate uterus, is essential because treatment strategies differ substantially [13,14]. Unlike a septate uterus, which is effectively treated by hysteroscopic septoplasty, a bicornuate uterus generally does not benefit from hysteroscopic correction. Therefore, careful preoperative anatomical assessment is fundamental to avoid inappropriate management [6,7,12,13]. Table 1 provides a summary of the differential diagnosis of congenital Müllerian anomalies, emphasizing the key distinguishing embryological, imaging, clinical, and management features that facilitate accurate diagnosis and guide appropriate clinical decision-making.

**Table 1:** Differential diagnosis of congenital Müllerian anomalies. Sources: adapted from ASRM Müllerian Anomalies Classification (2021), ESHRE/ESGE classification, and published reviews on congenital uterine anomalies [2-4,11]. 3D: three-dimensional; TVUS: transvaginal ultrasonography; MRI: magnetic resonance imaging; ASRM: American Society for Reproductive Medicine; ESHRE/ESGE: European Society of Human Reproduction and Embryology/European Society for Gynaecological Endoscopy

Anomaly	Embryological defect	Key imaging features (3D TVUS/MRI)	External fundal contour	Clinical presentation	Reproductive outcomes	Recommended management
Septate uterus	Failure of resorption of the midline Müllerian septum after normal duct fusion	Fibrous or fibromuscular septum dividing the endometrial cavity; normal uterine fundal contour	Normal or mildly indented (<1 cm)	Often asymptomatic; infertility, recurrent pregnancy loss, preterm birth, malpresentation	Increased risk of infertility, miscarriage, and adverse obstetric outcomes	Hysteroscopic septum resection is the treatment of choice in appropriately selected symptomatic patients
Bicornuate uterus	Partial failure of Müllerian duct fusion	Two separate uterine cavities with a deep external fundal cleft	Deep fundal indentation (>1 cm)	Recurrent pregnancy loss, preterm birth, malpresentation; infertility less common	Increased risk of miscarriage, preterm labor, and fetal malpresentation	Usually conservative; metroplasty reserved for selected patients with recurrent adverse reproductive outcomes
Arcuate uterus	Mild incomplete resorption of the uterine septum (normal anatomical variant)	Mild, smooth concavity of the endometrial cavity without significant cavity division	Normal	Usually asymptomatic	Minimal or no significant effect on fertility or pregnancy outcomes	No treatment required
Uterus didelphys	Complete failure of Müllerian duct fusion	Two completely separate uterine cavities with two cervices; may have longitudinal vaginal septum	Completely separated hemiuteri	Dysmenorrhea, dyspareunia, infertility (less common), recurrent pregnancy loss	Increased risk of miscarriage, preterm birth, malpresentation	Conservative management in most cases; surgical correction rarely indicated
Unicornuate uterus	Incomplete development of one Müllerian duct	Small, asymmetric banana-shaped uterus; may have rudimentary horn	Single hemiuterus	Infertility, recurrent miscarriage, preterm labor, ectopic pregnancy in rudimentary horn	Poor reproductive outcomes, fetal growth restriction, preterm birth	Individualized management; excision of functional rudimentary horn when indicated
Uterine agenesis (Mayer-Rokitansky-Küster-Hauser syndrome)	Complete or near-complete failure of Müllerian duct development	Absence or severe hypoplasia of uterus; normal ovaries	Absent uterus	Primary amenorrhea with normal secondary sexual characteristics	Natural pregnancy not possible; ovarian function usually preserved	Multidisciplinary management including reproductive counseling and psychological support

Hysteroscopic septum resection has become the standard minimally invasive treatment for symptomatic women with a septate uterus because it restores normal uterine cavity anatomy while avoiding abdominal surgery [15]. The procedure is associated with low complication rates, rapid postoperative recovery, and preservation of reproductive potential. Nevertheless, the impact of septoplasty on fertility outcomes remains an area of ongoing investigation [15,16].

Recent systematic reviews and meta-analyses have demonstrated that hysteroscopic septum resection is consistently associated with a reduction in miscarriage rates and improved reproductive outcomes in appropriately selected patients [5,6,10,17]. However, evidence regarding improvement in live birth rates and overall fertility remains less definitive. The international randomized controlled trial conducted by Rikken et al. reported no statistically significant benefit of routine septum resection for all women with a septate uterus, highlighting the importance of individualized patient selection rather than universal surgical intervention [10]. Conversely, multiple observational studies have reported favorable improvements in clinical pregnancy rates, live birth rates, and reduction in pregnancy loss following hysteroscopic septoplasty [9]. These differences likely reflect variations in patient selection, study design, septum characteristics, and duration of follow-up [7,9,10]. A summary of recent evidence on the diagnosis and management of septate uterus, including current guidelines, systematic reviews, meta-analyses, and key clinical studies, is presented in Table 2.

**Table 2:** Summary of recent literature on the diagnosis and management of septate uterus (2021-2026). ASRM: American Society for Reproductive Medicine; ESHRE: European Society of Human Reproduction and Embryology; ESGE: European Society for Gynaecological Endoscopy; CUME: Congenital Uterine Malformation by Experts; MRI: magnetic resonance imaging; 3D TVUS: three-dimensional transvaginal ultrasonography; ART: assisted reproductive technology

Study (year)	Study design	Study population	Objective	Key findings	Clinical relevance to present case
ASRM Clinical Practice Guideline (2024) [1]	Evidence-based clinical practice guideline	Women with septate uterus	Update recommendations for diagnosis and management	Recommends standardized diagnosis using contemporary imaging and individualized consideration of hysteroscopic septum resection in symptomatic women; emphasizes shared decision-making.	Supports the diagnostic approach and individualized surgical management adopted in the present case.
Noventa et al. [2]	Systematic review and meta-analysis	Women with septate uterus with or without hysteroscopic metroplasty	Evaluate reproductive and obstetric outcomes	Septate uterus was associated with poorer reproductive outcomes. Hysteroscopic metroplasty significantly reduced miscarriage rates and improved reproductive outcomes in appropriately selected patients.	Reinforces the rationale for surgical correction in women with infertility.
Galati et al. [3]	Systematic review and meta-analysis	Women undergoing hysteroscopic septum resection	Assess pregnancy, live birth, and miscarriage outcomes	Septum resection significantly reduced miscarriage rates and improved clinical pregnancy rates, although improvement in live birth rates remained less consistent across studies.	Supports current evidence-based management while acknowledging ongoing controversy.
ASRM Müllerian anomalies classification (2021) [4]	Evidence-based clinical practice guideline	Infertile women diagnosed with septate uterus	Determine the effect of hysteroscopic metroplasty on fertility	Demonstrated improved pregnancy outcomes following hysteroscopic metroplasty, particularly in women with infertility and recurrent pregnancy loss.	Supports hysteroscopic septoplasty as a fertility-preserving intervention.
Alcázar et al. Diagnostic Accuracy [5]	Systematic review and meta-analysis	Diagnostic imaging studies	Compare ultrasound modalities for diagnosing septate uterus	Three-dimensional transvaginal ultrasound demonstrated the highest diagnostic accuracy and was comparable to MRI.	Justifies the use of advanced imaging for accurate diagnosis.
Vitale et al. [6]	Systematic review	Women undergoing hysteroscopic septoplasty	Evaluate postoperative second-look hysteroscopy	Second-look hysteroscopy may facilitate early detection of residual septum or intrauterine adhesions in selected patients, although routine use remains controversial.	Highlights the importance of postoperative cavity assessment during follow-up.
Dixit et al. [7]	Systematic review	Women with complete septate uterus with cervical and vaginal septa	Review reproductive outcomes in complex anomalies	Complex septate anomalies were associated with poorer reproductive outcomes and frequently required individualized surgical management.	Emphasizes accurate classification before treatment planning.
Daniilidis et al. [9]	Narrative review	Literature review	Review diagnosis and treatment of septate uterus	Summarized current concepts regarding pathophysiology, diagnostic imaging, and hysteroscopic treatment strategies.	Provides supporting evidence for the Discussion section.
Grimbizis et al. [10]	Comparative diagnostic study	Women undergoing gynecologic imaging	Compare ASRM, ESHRE/ESGE, and CUME classification systems	Demonstrated differences in diagnostic thresholds among classification systems that may influence prevalence estimates and treatment decisions.	Highlights the importance of standardized classification during diagnosis.
Almandoz et al. [11]	Prospective diagnostic study	Women with suspected uterine anomalies	Evaluate the accuracy of 3D TVUS	Three-dimensional ultrasonography accurately identified uterine septa and allowed precise measurement of septal morphology.	Supports the imaging strategy used in this case.
Ramakrishnan et al. [12]	Comparative diagnostic study	Women with Müllerian anomalies	Compare MRI and 3D ultrasonography	Both modalities demonstrated excellent diagnostic performance; MRI remained valuable for complex anomalies.	Supports complementary use of MRI when diagnosis is uncertain.
Piriyev et al. [13]	Retrospective cohort study	Women undergoing hysteroscopic metroplasty	Evaluate surgical safety and reproductive outcomes	Demonstrated low complication rates, minimal adhesion formation, and favorable reproductive outcomes following hysteroscopic septoplasty.	Supports the safety profile of minimally invasive hysteroscopic surgery.
Kaveh et al. [14]	Clinical cohort study	Women undergoing ART after septoplasty	Evaluate ART outcomes following septum resection	Surgical correction before ART was associated with improved implantation and pregnancy outcomes in selected patients.	Relevant for counseling infertile women considering assisted reproduction.
Hurni et al. [15]	Retrospective cohort study	Women following hysteroscopic septum resection	Determine incidence of intrauterine adhesions	Intrauterine adhesions were uncommon after careful hysteroscopic septoplasty, although follow-up hysteroscopy was beneficial in selected high-risk patients.	Supports postoperative surveillance recommendations.
Elaitari et al. [16]	Case report	Complete septate uterus with double cervix and vaginal septum	Describe diagnosis and surgical management of a rare anomaly	Demonstrated successful management of a complex Müllerian anomaly using individualized surgical planning.	Highlights the spectrum of septate uterine anomalies and the need for accurate classification.
Nakamoto et al. [17]	Case report	Dichorionic diamniotic twin pregnancy with complete septate uterus	Report obstetric management of a rare pregnancy	Demonstrated that successful pregnancy is possible despite complex uterine anatomy with appropriate multidisciplinary care.	Illustrates the potential reproductive implications of untreated congenital uterine anomalies.

Complete excision of the septum appears to be an important determinant of successful reproductive outcomes [16]. Residual septal tissue may impair implantation and has been associated with persistent infertility or recurrent pregnancy loss. Consequently, careful intraoperative visualization and, in selected cases, postoperative assessment using three-dimensional ultrasonography or second-look hysteroscopy may optimize surgical outcomes, particularly among women undergoing assisted reproductive techniques [16-18].

The management of a septate uterus primarily relies on hysteroscopic correction (septoplasty or metroplasty), which has been shown to improve reproductive outcomes. However, as highlighted by Dason et al. [18], while numerous surgical variations exist, there remains a notable lack of high-quality, standardized evidence supporting one specific technique over another. Guidelines from the Society of Laparoscopic and Robotic Surgeons (SLS) underscore the importance of meticulous evaluation and careful technique to prevent intraoperative and postoperative complications during septum resection.

A primary concern following any hysteroscopic intervention is the formation of IUAs, which can negatively impact future fertility. Hafizi et al. [19] emphasized the necessity of evaluating post-surgical IUA formation, noting that endometrial healing plays a critical role in overall success.

The choice of surgical energy modality is central to this debate. Recent data from Piriyev et al. [13] evaluating electrosurgical hysteroscopic metroplasty demonstrated favorable perioperative safety profiles, though post-operative adhesion rates remain a key metric of long-term success. To minimize thermal damage and subsequent adhesion formation, many surgeons advocate for mechanical instruments. This is supported by broader hysteroscopic data; a 2021 meta-analysis in Medicine (Baltimore) [20] and a multicenter randomized controlled trial in the International Journal of Surgery comparing cold scissors to electrosurgery found that mechanical "cold" techniques (such as the cold scissors ploughing technique) significantly reduce postoperative adhesion scores and preserve surrounding healthy endometrium compared to electrosurgical excision (Table 3).

**Table 3:** Surgical instrument choice for hysteroscopic septum resection. Source: Piriyev et al. [13], Hafizi et al. [19], and Yang et al. [20]

Aspect	Cold scissors	Electrosurgery
Current majority use	Minority	~64% (majority)
Why chosen	Lower cost; no electrolyte-fluid limit	Standard OR equipment; familiar training
Adhesion rate (single-center studies)	23.3% [19]	4.3% [13]
Adhesion recurrence (pooled meta-analysis, n = 761)	Lower (OR 0.30, p = 0.0002)	Higher recurrence
Operation time	Shorter	Longer
Blood loss	Lower	Higher
Post-op menstrual flow	Better	Lower
Pregnancy/live birth rate	No significant difference	No significant difference
Patient pain during procedure	Lower (mild pain 66.7%)	Higher (moderate pain 73.3%)
Septum-specific head-to-head RCT	Comparable at 3 months	Comparable at 3 months
Proven superiority?	Not established	Not established
Guideline stance	Surgeon/institution preference	Surgeon/institution preference

Consequently, while electrosurgery remains a widely utilized and efficient approach for septum resection, the integration of cold scissors or anti-adhesion barriers should be strongly considered to optimize tissue healing and protect the patient's reproductive potential. The present case illustrates several important clinical principles. The patient presented with prolonged unexplained primary infertility despite normal hormonal evaluation and a normal partner assessment. Comprehensive anatomical evaluation ultimately identified a septate uterus as the underlying structural abnormality. Successful hysteroscopic septum resection restored a normal uterine cavity without perioperative complications, and postoperative imaging confirmed complete correction of the anomaly. This clinical course is consistent with current recommendations emphasizing the importance of evaluating uterine anatomy in women with otherwise unexplained infertility before proceeding to more invasive fertility treatments.

Although a septate uterus is a well-recognized congenital anomaly, published case reports and clinical data from the South Caucasus region remain limited. To our knowledge, detailed reports describing the diagnosis and hysteroscopic management of uterine septum in women with primary infertility from Azerbaijan are scarce. This case therefore contributes valuable regional clinical experience by demonstrating the successful application of contemporary diagnostic imaging and minimally invasive hysteroscopic management within a resource-variable healthcare setting.

In many developing and transitional healthcare systems, delayed diagnosis may occur because advanced imaging modalities such as three-dimensional transvaginal ultrasonography and magnetic resonance imaging are not uniformly available, and specialized reproductive medicine services may be limited [12]. Consequently, women with CUAs may undergo prolonged infertility evaluation before the underlying anatomical abnormality is identified. This case emphasizes the importance of improving access to specialized gynecologic imaging and infertility services to facilitate earlier diagnosis and appropriate intervention.

This case reinforces the importance of including CUAs in the differential diagnosis of women presenting with unexplained primary infertility, even when endocrine evaluation, ovulatory function, and male factor assessment are normal. Comprehensive assessment of uterine anatomy should be considered an integral component of infertility investigations because early identification of correctable structural abnormalities may reduce unnecessary delays in treatment and improve reproductive planning [9]. Hysteroscopic septum resection remains a safe, minimally invasive procedure that can effectively restore normal uterine anatomy with minimal perioperative morbidity.

The strengths of this report include comprehensive infertility evaluation, confirmation of the diagnosis using advanced imaging, successful minimally invasive surgical management, and postoperative documentation of restoration of a normal uterine cavity. Furthermore, the discussion integrates contemporary evidence and international guideline recommendations with regional clinical experience.

The principal limitation of this report is its single-patient design, which precludes generalization of clinical outcomes. In addition, long-term reproductive outcomes, including pregnancy and live birth, were not available at the time of manuscript preparation. Larger prospective studies with extended follow-up are needed to further clarify the impact of hysteroscopic septum resection on fertility and obstetric outcomes and to better define the patients most likely to benefit from surgical intervention.

## Conclusions

In summary, this case highlights the successful management of a 26-year-old nulligravid woman who presented with primary infertility of three years' duration attributed to a complete septate uterus. Prompt diagnosis utilizing three-dimensional transvaginal ultrasonography and accurate anatomical categorization facilitated targeted intervention via a hysteroscopic septum resection using cold scissors. Post-operatively, follow-up evaluation confirmed the restoration of a normal uterine cavity, and the patient completely recovered without any intraoperative or postoperative complications.

Ultimately, this report underscores the critical importance of utilizing standardized classification frameworks to guide appropriate anatomical correction in congenital Müllerian anomalies. Bridging the gap between precise radiological assessment and definitive, minimally invasive clinical execution remains paramount for restoring normal uterine anatomy and optimizing future reproductive outcomes. Furthermore, increased clinical awareness and timely access to advanced diagnostic imaging are essential, particularly within resource-variable healthcare settings where these correctable structural anomalies may otherwise remain underdiagnosed.
